# Recruitment to Online Therapies for Depression: Pilot Cluster Randomized Controlled Trial

**DOI:** 10.2196/jmir.2367

**Published:** 2013-03-05

**Authors:** Ray B Jones, Lesley Goldsmith, Paul Hewson, Christopher J Williams

**Affiliations:** ^1^Plymouth UniversityFaculty of Health, Education, and SocietyPlymouthUnited Kingdom; ^2^Plymouth UniversityDepartment of Maths and ComputingPlymouthUnited Kingdom; ^3^University of GlasgowInstitute of Health and WellbeingGlasgowUnited Kingdom

**Keywords:** cluster randomized trial, pilot study, online advertising, depression, MoodGym, Living Life to the Full, LLTTF, cCBT, Google Analytics, AdWords, computerised CBT

## Abstract

**Background:**

Raising awareness of online cognitive behavioral therapy (CBT) could benefit many people with depression, but we do not know how purchasing online advertising compares to placing free links from relevant local websites in increasing uptake.

**Objective:**

To pilot a cluster randomized controlled trial (RCT) comparing purchase of Google AdWords with placing free website links in raising awareness of online CBT resources for depression in order to better understand research design issues.

**Methods:**

We compared two online interventions with a control without intervention. The pilot RCT had 4 arms, each with 4 British postcode areas: (A) geographically targeted AdWords, (B) adverts placed on local websites by contacting website owners and requesting links be added, (C) both interventions, (D) control. Participants were directed to our research project website linking to two freely available online CBT resource sites (Moodgym and Living Life To The Full (LLTTF)) and two other depression support sites. We used data from (1) AdWords, (2) Google Analytics for our project website and for LLTTF, and (3) research project website. We compared two outcomes: (1) numbers with depression accessing the research project website, and then chose an onward link to one of the two CBT websites, and (2) numbers registering with LLTTF. We documented costs, and explored intervention and assessment methods to make general recommendations to inform researchers aiming to use similar methodologies in future studies.

**Results:**

Trying to place local website links appeared much less cost effective than AdWords and although may prove useful for service delivery, was not worth pursuing in the context of the current study design. Our AdWords intervention was effective in recruiting people to the project website but our location targeting “leaked” and was not as geographically specific as claimed. The impact on online CBT was also diluted by offering participants other choices of destinations. Measuring the impact on LLTTF use was difficult as the total number using LLTTF was less than 5% of all users and record linkage across websites was impossible. Confounding activity may have resulted in some increase in registrations in the control arm.

**Conclusions:**

Practitioners should consider online advertising to increase uptake of online therapy but need to check its additional value. A cluster RCT using location targeted adverts is feasible and this research design provides the best evidence of cost-effectiveness. Although our British pilot study is limited to online CBT for depression, a cluster RCT with similar design would be appropriate for other online treatments and countries and our recommendations may apply. They include ways of dealing with possible contamination (buffer zones and AdWords techniques), confounding factors (large number of clusters), advertising dose (in proportion to total number of users), record linkage (landing within target website), and length of study (4-6 months).

**Trial Registration:**

clinicaltrials.gov (Registration No. NCT01469689); http://clinicaltrials.gov/ct2/show/NCT01469689 (Archived by WebCite at http://www.webcitation.org/6EtTthDOp)

## Introduction

Less than 60% of people with diagnosable depression or anxiety seek formal help from practitioners; this represents a significant treatment gap [1]. The remainder may access informal care, alternative therapies, make private arrangements such as counselling, use the voluntary sector, the Internet, or use other sources of information. Nearly 1 in 5 British Internet users search for information related to mental health [2], but patients searching for health information online may not find what they are looking for [3], possibly due to sub-optimal search strategies [4].

There is increasing evidence that online interventions can be effective in changing health behaviors or improving health [5]. Online cognitive behavioral therapy (CBT), is an example offering effective self-help treatment for depression. Online CBT is recommended by the National Institute for Health and Clinical Excellence (NICE) in the UK for mild to moderate depression [6]. The range of resources includes licenced (paid for) sites such as Beating the Blues [7], and free access websites providing access to CBT life skills resources (eg, Living Life To The Full (LLTTF) [8] and MoodGYM [9]).

However, many patients do not benefit through lack of awareness. We found a variation in registration to LLTTF of 15-fold between the highest (Kirkwall, Scotland) and lowest (Wigan, England) postcode areas [10]. Variations in Internet use by region in Britain are small; 80.3% of those in the North East had at some time accessed the Internet compared to 87.6% in London by 2012 [11]. Variation in the prevalence of depression between postcode areas is small [12], and although other packages such as MoodGym may be in use, the most likely explanation for variation in LLTTF registration was lack of awareness.

Raising awareness of online CBT could benefit many people with depression by facilitating early rapid access. Ways of addressing this include online advertising through search engines such as Google AdWords (AdWords) [13-19], advertising on social media sites [20], “snowballing” on social media sites [21], getting websites of other organizations to add links (weblinks) [22], using offline via mass media [23], or via practitioners [24].

AdWords have been used by others to recruit participants to studies, for example, for depression screening [25], use of condoms [26], and quit smoking campaigns [27], but the cost effectiveness of their use has not been assessed. Weblinks from a range of existing sites have also been used in research studies, often routinely as part of a “recruitment package” (eg, [22] and LLTTF is linked from sites such as the English National Health Service’s (NHS) website NHS Choices [28]), but we were unaware of any study of weblinks restricted to local organization websites in order to target recruitment on a specific local population as required for this design.

Studies that compare recruitment methods before and after interventions have no control group that could be described and compared with adequately. The only rigorous way of comparing methods of raising awareness of online therapies is by geolocated cluster randomised controlled trials (RCT, [10]). Matching intervention and control areas reduces the chances of bias. If we can limit online advertising to one geographic area and compare it with another area where there is no advertising, we can then assume that any difference is due to the advertising. We can therefore estimate its cost effectiveness in terms of cost per new user. Subsequently, we can decide if it is worth using online advertising to raise awareness, or whether other methods would be more cost effective.

We planned to compare purchasing AdWords with the second strategy of using weblinks and carried out a pilot RCT of the two different recruitment interventions to check the methods and outcome measures for a definitive trial later. In particular, our study objectives were to explore: (1) whether or not the two recruitment interventions seemed to work at all, and so be worthy of further study, (2) the ability to target online Google adverts or weblinks from other local websites without contamination, (3) the ability to link data sources to effectively measure the impact of the recruitment interventions, and (4) to learn more about the likely size and impact on target CBT site use and, given the other methodological issues such as confounding factors, to know what sample size and dose of advertising will be needed for future substantive studies.

## Methods

### Ethics and Registration

The study was approved by the NHS South West 2 Research Ethics Committee (Reference 11/H0203/8; February 2011) and registered on clinicaltrials.gov (Registration No. NCT01469689).

### Design

**Figure 1:**
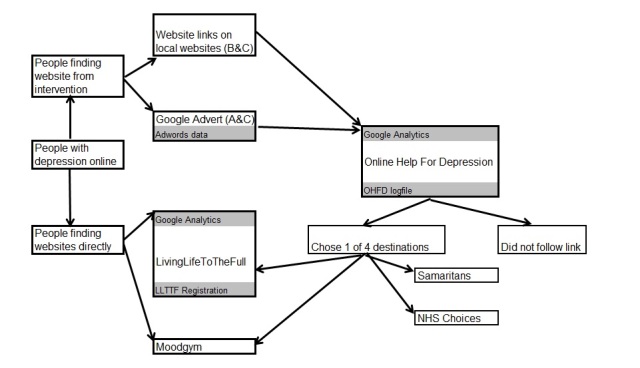
Schematic of the design showing websites and data sources (in gray).

This was a pilot cluster RCT of recruitment interventions for online CBT for depression. We compared two online recruitment interventions with a control without intervention. The pilot RCT had 4 arms: (A) geographically targeted paid AdWords, (B) free adverts/weblinks placed on local websites by contacting those website owners (weblinks), (C) both interventions combined, (D) control. Participants were directed to a project recruitment website linking to two freely available online CBT sites (Moodgym and LLTTF) and two other sites (Samaritans [29] and NHS Choices [28]). We used data from (1) AdWords, (2) Google Analytics (Analytics) for our project website and for LLTTF, and (3) our Online Help For Depression (OHFD) project website. We examined 2 outcomes in the 4 arms of the study: (1) numbers accessing the project recruitment website that had depression, and then chose an onward link to one of the two CBT websites, and (2) numbers registering with LLTTF. We documented costs and explored intervention and assessment methods to make recommendations for a definitive trial, for other researchers carrying out similar studies, and tentative recommendations for practitioners and policy makers. Figure 1 shows a schematic of the design.

### Sampling and Randomization

All 121 postcode areas in England, Wales, and Scotland were divided into quartiles by rate of registration on LLTTF (based on 36,753 registrations of people between June 2008 and June 2009 who scored 8 or more on the Hospital Anxiety and Depression Scale for either anxiety or depression) and into quartiles by population size (based on the 2001 census). We randomly selected 4 cells from this array, and chose 4 nearly consecutive postcode areas for each cell (to try to achieve similar populations), avoiding adjacent geographical areas. Each set of 4 postcode areas was randomly allocated to the 4 arms of the trial (three interventions and control). Randomization was not blind.

### Sample

Each arm of the study had a total population ranging from 1.6 to 2 million people clustered in 4 postcode areas. In total the study included 7 million people in 16 postcode areas across England, Wales, and Scotland. The estimated point prevalence for major depression among 16- to 65-year olds in the UK was 2.1%, rising to 9.8% when the less specific and broader category of “mixed depression & anxiety” was included [30]. We used an intermediate estimate of the prevalence of depression of 5%, which gave an estimated target population of 350,000.

### Interventions

Those paying for AdWords campaigns set up one or more adverts, and enter keywords to help determine when the advert is shown. AdWords displays adverts as a sponsored link, either at the top of the list of search results or in the right hand search results panel, depending on the phrase entered, the price offered per advert, bids from competing adverts, and (if requested) by estimated location of the user.

Within the 8 areas in arms A and C, we ran AdWords from April 17^th^ to November 30^th^, 2011. In the 8 areas in B and C, we aimed to place adverts (weblinks) from local organization websites such as local universities, general practitioner (GP) practices, and local authorities, by contacting these organizations via email and/or phone starting on April 17^th^. Arm D was a control arm with no recruitment intervention.

### Google AdWords

**Figure 2:**
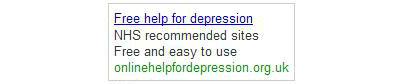
Google Advert.

We used a single advert (Figure 2) from April 17^th^ to October 19^th^ as one campaign with a daily budget of £7.50 per day (4.4 pence per 1000 target population per day). Targeting specific postcode areas (eg, Kingston, KT) was not an option offered by AdWords. Options did however include targeting a radius of 1 mile or more around a postcode district (eg, KT2) or to hand draw a polygon to enclose the area of interest. We used a mix of methods: 4 postcode areas were defined using circles of 1 mile radius for all postcode districts within the postcode area and 4 were defined by hand drawn polygons. Preliminary analysis [10] showed leakage from the target areas and an imbalance in the number of presentations of the advert between postcode areas. This prompted a change of strategy with 8 separate adverts for each postcode area and adverts that mentioned the target area. Radius targeting (rather than polygon targeting) was used for all areas from October 19^th^ to November 30^th^. The daily budget was increased to £16.10 (9.3 pence per 1000 target population per day) and divided between the 8 areas in proportion to target population.

We originally asked AdWords to display the advert for the keyword of depression. AdWords suggested other similar keyword combinations and we accepted all suggestions. AdWords gave information on the number of times they presented adverts against Google searches, by day, keyword, and location. AdWords decided when to present the advert based on the price we offered, the price of competing adverts, and other factors such as the search terms used. Users searching on terms such as depression and depression help were presented with our advert, depending on our budget and competing adverts.

### Local Weblinks

Google searches were used to identify organizations in 8 postcode areas (arms B and C) with websites to place adverts (weblinks to our project website) on local free access websites. In our search we included websites such as local GP surgeries, local media websites including newspaper, TV, radio, further and higher education institutions, and community-based or local charity websites. In total, 180 emails were sent with 3 weblinks posted free of charge: two in Leeds (university medical practice and a carers’ organization), and one in Kirkwall (local online community newspaper).

### Project Website

Those who clicked on Google adverts or on weblinks were directed to our project research website OHFD. This gave information about the study and advised that completion of the online questions implied consent for it to be used in the study (see Multimedia Appendix 1 for screenshots). Computer Internet Protocol (IP) addresses, dates, and times were collected via OHFD, and also monitored by Analytics. We specifically did not try to raise the visibility of our website to normal Google, Yahoo, or Bing search engines. Visitors were asked for their postcode area and to complete the Patient Health Questionnaire (PHQ9) [31] assessing depression. Users were then offered 4 links to Moodgym, LLTTF, NHS Choices information on depression, and Samaritans. The order in which the links to Moodgym and LLTTF (top row) and NHS Choices and Samaritans (bottom row) appeared was randomized within row.

### Data Sources

We used various sources of data to model patient flow (Figure 1). AdWords and Analytics were used for OHFD. The website log for OHFD recorded stated postcode areas from participants and their website destination choice (if made). Analytics was used for all visitors of LLTTF, identifying those referred by OHFD and those likely to be in study arms (using Google defined locations). Website log was used for LLTTF, including those who registered and stated their postcode area.

### Costs

We documented costs for using AdWords and other weblinks and estimated costs per person with depression referred to the online CBT via AdWords, based on time spent, cost £10/hour (based on the hourly rate Plymouth University pays temporary administrative staff) and Google’s charges. We included costs needed for routine delivery of these methods and excluded research costs such as time spent in the comparison of methods or in setting up the research project website.

### Outcomes

We defined 2 main outcomes and compared them between the 4 arms: (1) the numbers accessing the project research website (OHFD) that completed a PHQ9 depression rating score and had a score of more than 5, indicating at least mild depression, and then chose an onward link to one of the two CBT websites, and (2) the numbers registering with LLTTF who gave their postcode, comparing intervention with control and all areas. In the revised version of LLTTF (issued in January 2011) used in this study, new arrivals at LLTTF do not have to immediately register, instead, registration can be delayed. Registration, by giving more personal details and agreeing to email reminders, signifies a commitment to use the site seriously. In the previous version of LLTTF used to select the sample, most visitors to the site registered as they could not access most content until after registration.

### User Panel

We carried out this study at a time when the Improved Access to Psychological Therapies (IAPT) project [32] was investing large amounts of money to improve access to psychological therapies across England. To try to identify other local initiatives and how these may have affected our sample and interventions, we aimed to recruit a panel of informants in our study areas. We emailed five ex-users of LLTTF from each of the 16 areas (ie, total 80), and also tried to contact them via IAPT teams. Panel members were to be paid £30 at the end of the study by e-vouchers.

## Results

### Did the Two Interventions Work at All?

**Figure 3:**
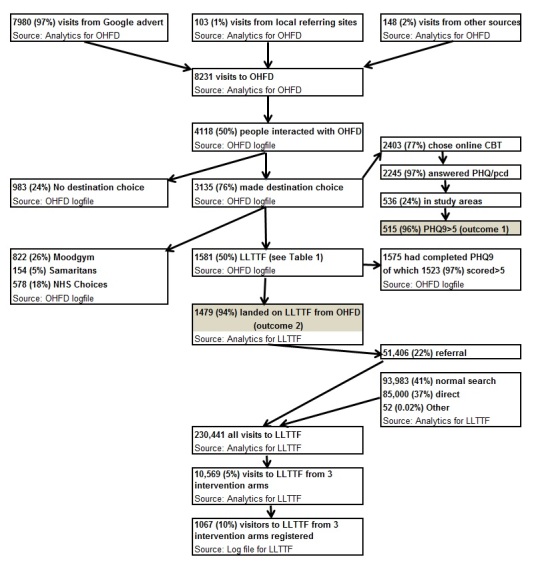
Participant flow diagram showing overall recruitment and different data sources (April 17^th^ - November 30^th^ 2011). Shaded boxes show numbers for the two outcomes.

Examining the number of people clicking through from online adverts to OHFD to online CBT (Figure 3), we saw that our OHFD research site had a total of 8231 visits, the majority (97%, 7980/8231) from Google adverts and only 1% (103/8231) from weblinks. Half of those who visited OHFD (50%, 4118/8231) interacted with the site and 76% (3135/4118) made a choice of destination from the 4 available sites, three-quarters of whom (77%, 2403/3135) chose the online CBT. Although 96% (2306/2403) had depression (according to the PHQ9 score >5), only 515/2306 (22%) were from the study target areas (Table 1).

Table 1 shows that the rates of referral to online CBT for AdWords arms A and C were higher than the weblinks only arm B (450 and 387 vs 93, respectively, per 100,000) and much higher than the control arm (D) (28 per 100,000). These results were confirmed by fitting a Poisson generalized linear model with the R software [33], with predictors for the use of AdWords, weblinks, and an interaction term for both. This produced a 95% confidence interval for rates relative to control of (10.2 and 27.4, respectively) when using Google adverts. The rate relative to control for weblinks was (2.0 and 6.0, respectively) and there was little to be gained from using both, given an interaction between AdWords and weblinks with rate relative to baseline of (0.1, 0.5). The majority of those choosing online CBT chose LLTTF (66%, 1581/2403) and the difference between arms A/C and B/D was still evident (Table 1).

### Was it Possible to Link Data to Measure Outcome Two?

It proved impossible to directly track individuals from OHFD to LLTTF using IP address and time data, therefore the impact of the interventions on LLTTF had to be assessed using Analytics and LLTTF website data. Analytics reported 1474 visits landing on LLTTF from OHFD, agreeing approximately with the 1581 referred by OHFD, although the 7% attrition is unexplained (Figure 3). During the period of study, according to Analytics, the majority of the 230,441 visits to LLTTF were from normal search (41%, 93,983/230,441) or direct (37%, 85,000/230,441), with 22% (51,406/230,441) from referring sites. In total, there were 1888 sites from which there were 51,406 visits. Of the referring sites, OHFD was the seventh largest referrer with 1474 but representing just 2.9% (1474/51,406) of referrals and less than 1% (1474/230,441) of all visits. The Royal College of Psychiatrists sent most referrals (5071/51,406, 9.9%), but this still represented only 2.2% (5071/230,441) of all visits.

We know from Analytics that the bounce rate (ie, those who exited from the first page) for visits from OHFD was 47% (no different to the average bounce rate from referred visits of 49%). This suggests that those arriving from OHFD were not more or less likely to continue and to subsequently register. We can see from the difference between arrivals on the site (14,396 from Analytics) and registrations (1143 from LLTTF log data) from the study arms that 10% (1067/10569) of all visitors registered on the current version of LLTTF.

Table 1 shows that the 95% confidence intervals of rate of registration on LLTTF (outcome 2) overlap between all 4 arms, in other words there was no significant difference between the interventions or control. Although none of the differences for individual postcode areas was significant, registration rates for most (11/16, 69%) postcodes tended to decline from January-April to April-November. Of the more populous postcode areas, Liverpool (arm A) showed the greatest increase (360 to 489, 26% increase) but this was still not significant and Nottingham (in the control arm D) had a 6% increase. Two of the small Scottish island postcode areas may have increased but their populations are small, the confidence intervals on estimates are large, and the impact on the whole arm small.

**Table 1:** Number of people on OHFD with depression (PHQ>5) choosing online CBT and LLTTF, registering on LLTTF, annual rate per 100,000 estimated depressed registering on LLTTF before and during interventions, by postcode area, and trial arm.

			Outcome 1 People on OHFD with depression choosing online CBT and LLTTF			Outcome 2 People registering on LLTTF before the intervention (Jan-Apr 2011) and during the intervention (Apr-Nov 2011)			
			Intervention period Apr-Nov 2011			Before intervention Jan-Apr 2011		Intervention period Apr-Nov 2011	
		Estimate of people with depression	People with depression who chose online CBT	Annual rate per 100,000 depressed (95% CI)	People with depression who chose LLTTF	Reg^a^ LLTTF	Annual rate per 100,000 depressed (95% CI)	Reg^a^ LLTTF	Annual rate per 100,000 depressed (95% CI)
**Arm A**									
	Liverpool (L)	42,173	155	588 (495-681)	111	38	360 (246-475)	129	489 (405-574)
	Redhill (RH)	24,721	49	317 (228-406)	34	59	955 (711-1198)	94	608 (485-731)
	Lancaster (LA)	16,299	42	412 (288-537)	27	44	1080 (761-1399)	73	717 (552-881)
	Harrogate (HG)	6668	7	168 (44-292)	6	19	1140 (627-1652)	40	960 (662-1257)
	Total	89,860	253	450 (395-506)	178	160	712 (602-823)	336	598 (534-662)
**Arm B**									
	Leeds (LS)	36,867	24	104 (62-146)	16	69	749 (572-925)	171	742 (631-853)
	Southend (SS)	24,660	12	78 (34-122)	8	55	892 (656-1128)	130	843 (698-988)
	Slough (SL)	16,882	10	95 (36-154)	6	22	521 (303-739)	59	559 (417-702)
	Kirkwall (KW)	2505	1	64 (-61-189)	0	17	2715 (1424-4005)	30	1916 (1230-2602)
	Total	80,914	47	93 (66-120)	30	163	806 (682-929)	390	771 (695-848)
**Arm C**									
	London (SW)	39,167	104	425 (343-506)	77	86	878 (693-1064)	170	694 (590-799)
	Kingston (KT)	24,505	72	470 (362-579)	50	36	588 (396-780)	75	490 (379-601)
	Darlington (DL)	17,074	21	197 (113-281)	15	37	867 (588-1146)	78	731 (569-893)
	Shetland (ZE)	1099	1	146 (-140-131)	1	5	1819 (225-3414)	18	2620 (1409-3830)
	Total	81,846	198	387 (333-441)	143	164	802 (679-924)	341	667 (596-737)
**Arm D**									
	Nottingham (NG)	54,012	10	30 (11-48)	8	102	755 (609-902)	272	806 (710-902)
	Oldham (OL)	22,190	3	22 (-3-46)	1	42	757 (528-986)	69	498 (380-615)
	Dudley (DY)	19,882	4	32 (1-64)	4	30	604 (388-820)	56	451 (333-567)
	Hebrides (HS)	1325	0	0 (0-0)	0	4	1207 (24-2391)	15	1811 (895-2728)
	Total	97,409	17	28 (15-41)	13	178	731 (624-838)	412	677 (611-742)
Study total		35,0028	515	235 (215-256)	364	665	760 (702-818)	1479	676 (642-711)
Other areas		25,04345	1791	114 (119-120)	1130	5613	897 (873-920)	11894	760 (746-773)
E, W, and S		28,54373	2306	129 (124-135)	1581^b^	6278	880 (858-902)	13,373	750 (737-762)

^a^registrations

^b^1581 includes 87 who gave no postcode on OHFD, but all other indications (eg, IP address) show that they were England (E), Wales (W), or Scotland (S).

### Validity Check on Location

As a validity check on location, we carried out an alternative analysis on those registering on LLTTF using the Analytics location data instead of user stated postcodes. The two approaches showed agreement.

### Possible Confounding Factors

**Figure 4:**
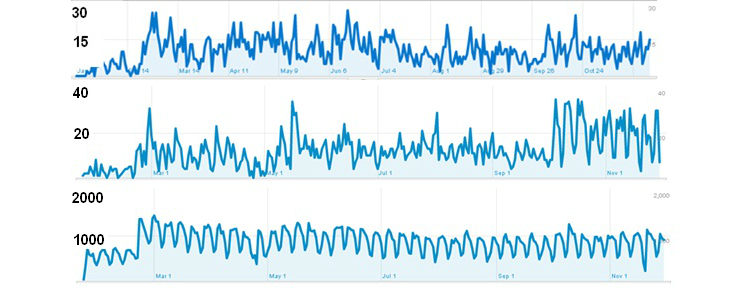
Number of visits to LLTTF according to Google Analytics. Top graph shows Liverpool, middle Nottingham, and the bottom graph shows ALL UK visits between January and November 2011.

According to Analytics, the biggest increase in “landings” on LLTTF was seen in one of the control areas, Nottingham. Figure 4 (middle panel) suggests that there may have been a “step change” for Nottingham in October 2011. Overall, the numbers visiting LLTTF were largely unchanged over the study period (apart from the very regular weekly cycle of visits with fewer during weekends, Figure 4 bottom panel).

### Size of the Impact on LLTTF

**Figure 5:**
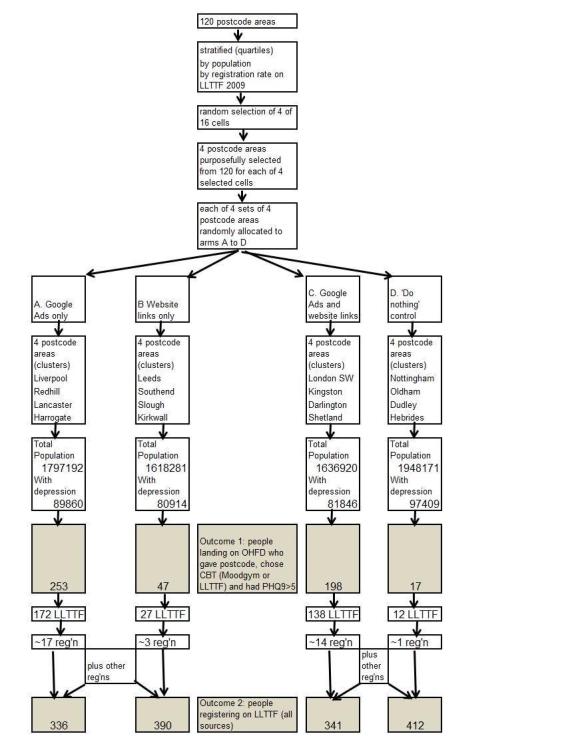
Participant flow diagram showing randomization to the 4 arms of the study.

Ignoring the stated locations of those clicking through to LLTTF, we see that 1581 clicked through to LLTTF in the study period (Figure 3). In the study period, the 3 intervention arms had 10,569 visits (estimated from Analytics) but only 1067 people registered, that is, with the current method of counting registration, only 10% of visitors to LLTTF registered. So if we estimated that, overall,10% of those referred from OHFD registered, we can see that the numbers referred from OHFD (for example, 17 in Arm A, Figure 5) were likely too small to greatly influence the total numbers registering in each of the trial arms (Figure 5, outcome 2-people registering on LLTTF). As we were unable to link individual participants from the project website (OHFD) to LLTTF, we could only tell if there had been an impact on the number of people registering on LLTTF if it was of sufficient size. If it had been possible to follow through individual participants from clicking on an advert to registering on LLTTF, having an impact on the total number registering from each area would have been less important.

### Leakage

Just under 8000 (7980/8231, 97%, Figure 3) visits to OHFD came from Google adverts, of which 4118/7980 (52%) interacted with OHFD, 3135/4118 made a destination choice, but only 515/3135 (16%) were from study arms with PHQ9 >5 who chose either Moodgym or LLTTF. Of these, only 387/4118 (9.3% of those who interacted with OHFD) chose LLTTF. Of those choosing LLTTF, probably only 10% registered, ie, less than 0.5% of those who clicked on the advert were likely to have registered on LLTTF. The biggest sources of “leakage” were from the location targeting [10] and from participants not engaging fully on LLTTF. The leakage however did not lead to much contamination of control areas. Failing to continue with the OHFD website and being lost to other or no Web destination also caused substantial leakage.

### Cost

All 3 weblinks were for locations in arm B. Arm C therefore was effectively another AdWords arm. The total cost of the AdWords campaign in payments to Google was £1841. From April 17^th^ to October 19^th^, we set a daily budget of £7.50 (4.4 pence per 1000 population) and had one campaign in which all 8 areas were included. As we described elsewhere [10], there was a disproportionate spending on AdWords for London SW. To counter this and to better understand the responses in each area, on October 19^th^, a new campaign in which each postcode area had its own budget was started. For the remaining 6 weeks, we increased the daily budget to £16.10 (9.4 pence per 1000 population), and divided this in proportion to the target population in each area (but giving a minimum budget of 60 pence for Shetland). Table 2 shows that the average number of clicks per day in the last 60 days was 29.3 (1759/60) compared to 34.2 (6291/184) in the first 184 days. This method (restricting the spending on London SW) also resulted in a higher cost/click overall, although we had noted in the first period that it takes AdWords 3-4 weeks to gain the optimum return on advertising spending after starting a new campaign.

We estimated that the time spent simply adjusting the AdWords campaign, as opposed to time spent on Analytics trying to match with other data, was 25 hours. At £10/hour, the total cost of the AdWords campaign was £2091 (£1841+£250). If we assume that both arms A and C were AdWords, this represents £4.64 per person for the 451 people who chose online CBT and had a PHQ9 >5 in arms A and C (the target group, Figure 5). Seventy hours were spent trying to contact owners of local websites to set weblinks, which if set at £10/hour, gives a total cost of £700 for arm B. This represents £14.89 per person for the 47 people in arm B (Figure 5).

**Table 2:** Payments to Google for AdWords campaign.

Area		Maximum daily budget		Average daily spend		Clicks		Cost	Cost per click
**April 17^th^** **-October 19^th^** **(184 days) 2011 single campaign**									
	All		£7.50		£7.07		6291	£1,301.04	£0.21
**8 individual campaigns by area October 20^th^-November 30^th^ (60 days)**									
	Liverpool		£3.84		£2.37		365	£142.45	£0.39
	London SW		£3.56		£2.48		886	£148.83	£0.17
	Redhill		£2.24		£1.19		182	£71.65	£0.39
	Kingston		£2.22		£1.43		174	£85.99	£0.49
	Darlington		£1.56		£0.76		66	£45.72	£0.69
	Lancaster		£1.48		£0.68		64	£40.61	£0.63
	Harrogate		£0.60		£0.08		21	£4.60	£0.22
	Shetland		£0.60		£0.00		1	£0.29	£0.29
	Total		£16.10		£9.00		1759	£540.14	£0.31

### User Panel

In total, we were able to recruit 12 panel members and all arms, but not all postcode areas, of the study were represented by either an ex-user of LLTTF or a member of an IAPT team. No other local interventions were identified via the user panel but the panel was able to help in examining the search environment across Britain [34].

## Discussion

### Need for the Study

McCrone estimated the number of people with depression in England as 1.24 million (2.3%) with total cost of services at £1.7 billion while lost employment increased this total to £7.5 billion [35]. Others estimated that a third of people with depression are not in contact with services [36]. Online CBT has a significantly small-to-medium effect size compared to non-active controls for patients with a range of severity of depressive symptoms and was recommended by NICE for mild to moderate depression [6]. There is virtually no extra cost in additional people using online CBT websites such as LLTTF but there was evidence that many people with depression who might benefit from using online CBT were missing the opportunity of effective treatment simply through lack of awareness. By raising awareness of its availability directly to depressed people, we could reduce inequalities in access to this treatment.

Various ways of raising awareness of websites are available including online advertising and weblinks. We did not know if online adverts such as AdWords would be more or less effective than weblinks. These methods may simply attract the same people that would in any case have found online CBT. As a pilot study, we were looking at the feasibility of being able to answer these questions and what study design would enable us to do so.

### Which Interventions Seemed to Work?

We have found that using AdWords is possibly going to be cost effective, whereas trying to get weblinks is not worth pursuing further in this locality based research study design. We only managed to put 3 weblinks in place. Scaling up the AdWords campaign would incur further advertising but no further labour costs (so the unit price gradually decreases), whereas scaling up the weblinks campaign would be proportional to the labour costs. Establishing weblinks therefore appears much less cost effective than AdWords and is not worth pursuing as a sole intervention in this study design.

However, the ability to establish weblinks may be significantly easier for local organizations trying to offer local services. In our study, we chose areas remote from the research team, and there were no prior local relationships on which to build. Being locally situated and delivering local services to local people might significantly affect the ease of establishing local websites. Others have used weblinks to successfully recruit as part of their recruitment package. For example, 91% (174/191) of participants in a trial of online treatment for chronic headache learned of the study from weblinks (both mutual and paid for) on websites, registration with major search engines, and notices posted to headache-related news groups, but the authors did not compare these methods and the study was global [22]. Overall 1 in 5 come to LLTTF from a weblink and various national website including NHS Choices and Royal College of Psychiatrists already have links to online CBT [34] and other nationwide charity websites may be willing to include weblinks. However, pursuing local websites such as primary care health centres to fit with this geo-targeted research design did not seem cost-effective. Nevertheless, this should not be discounted as an option for practitioners where it is known that weblinks can be put in place with little effort.

### Design Issues to be Addressed in a Definitive Trial

#### Overview

AdWords were effective in recruiting people to the project website but our pilot study identified a number of issues for our, and similar, cluster RCTs. The main problems were leakage so that its effect was greatly diluted, problems in linking data across websites so that the impact of advertising was lost in the large numbers using LLTTF, and possible confounding factors. The dose of advertising (particularly given the “record linkage” problem) and length of study also need to be considered. The changes that would be needed to the design of a definitive trial are discussed below. These findings will be relevant to others seeking to improve the uptake of online interventions or designing online cluster RCTs.

#### Leakage

There was leakage at 3 points in the process. First, we have shown elsewhere [10] that there was leakage from the target areas, particularly into neighbouring areas. This was partly due to the methods used and could be improved by using an area that is within the radius of set distance around a point rather than hand-drawn polygons, use of more appropriate radius, and avoidance of the area “edge”. Second, there was leakage caused by offering too many choices of destination to participants. We had included both MoodGym and LLTTF as the two main free online CBT sites as it was thought that there might be geographic variation in choice of online CBT. For ethical reasons we added Samaritans as another option, and added NHS Choices to create a balanced (even) number of options. As a result, only 50% of those making a choice chose LLTTF. As we did not have access to Moodgym log data, we were only able to follow participants to the point of choosing online CBT, and the impact on one of those sites (LLTTF). Third, with the current system of registration on LLTTF, only 10% of those accessing LLTTF decided to register and enter demographic data including their postcode (LLTTF allowed people to use the website without registering; registering offered further features such as email reminders).

To decide whether a definitive trial will be feasible, we modelled the effect of reduced leakage. By taking 50% of the number lost on the project website before making a destination choice, by routing all participants to LLTTF, and by reducing losses from location targeting from 76% to 20%, we would reduce the leakage from 94% to 47%.

#### Record Linkage and Insufficient Dose

We were not able to track individuals from OHFD to LLTTF and therefore could only use the overall data to try to estimate outcome 2, the number of people registering with LLTTF. Although outcome 1 showed significant differences between intervention and controls, to be able to measure a difference, the number referred from AdWords needed to be sufficiently larger compared to the total number that registered on LLTTF. The number of people with depression referred from OHFD to LLTTF was 4.7% (498/10569) of all users of LLTTF in study arms. If, as above, leakage was reduced to 47% the number referred by OHFD would still only be 10% of all those landing on LLTTF. To be certain that an impact of AdWords can be seen, the dose of advertising also needs to be increased. Although we doubled the daily budget to 9.3 pence per 1000 target population for the last two months of this pilot study, the number of clicks was slightly less than in the first period, probably due to breaking the advertising into 8 separate campaigns and AdWords not having had time “to settle”. The effectiveness of the AdWords advertising budget depends on competing adverts, and is likely to be less cost effective as the daily budget is increased. As shown in Table 2, the average daily spend was short of the maximum budget so it is not clear if an increase in daily budget would be spent or would increase the number of clicks, but a further doubling of the advertising budget would be worth trying.

#### Confounding Factors

A major contributing factor to the lack of impact on outcome 2 was the increase in uptake of LLTTF in one of the control areas (Nottingham), possibly as a result of a presentation given to practitioners by one of the authors (CW) to a national conference that overlapped with a surge of use. Although it was not a blinded region selection, day-to-day management of the study was conducted by RJ. We recognized in designing the pilot study that random interventions or effects from other influences, such as local campaigns, could impact on intervention or control areas. For that reason, we had attempted to monitor all areas via the user panel. This pilot study suggested that, to avoid an overdue influence of one postcode area (cluster), each arm needs many more than 4 clusters. As we had designed the pilot study with 2 types of interventions and 4 arms, we only used 16 out of 120 postcode areas for our study.

A more robust approach would be to use all postcode areas in Britain, excluding London postcodes, ordered by population size only, randomized in pairs to two arms, excluding any adjacent postcode areas as buffer zones around study areas. Using this method produces a two-arm study with 32 postcode areas (see Multimedia Appendix 2) and population of approximately 10 million in each arm. One arm would then be randomized to the intervention and one to the control group. The largest postcode area in one arm (Birmingham) still represents 17% of the total arm, so there is still some danger that other activity might lead to confounding factors, however this is much reduced from the design we used in this pilot where Nottingham represented 55% of the control arm population. This sample (Multimedia Appendix 2) of postcode areas could be adopted for other studies in Britain.

### Other Changes to Improve the Design of the Definitive Trial

#### Length of the Study

We paid for over 8 months of advertising but the recruitment numbers stabilized after a few months and we did not change the advert for 6 of the 8 months. On the other hand, it takes 2-3 months for AdWords to reach peak efficiency. With reduced leakage, a bigger sample, and an increased daily budget on advertising, the cost of a definitive study could probably be reduced by examining changes over 6 months.

#### Need for Better Methods of Assessing Location

Assessing location is subject to error. Previously [10], we have given a detailed comparison of user-reported versus methods based on IP address including using IP lookup tables and Analytics. The method we used to ask participants for their postcode area on our project website (OHFD) was probably the most accurate. We had hoped to be able to use IP addresses and the time of referral from OHFD to LLTTF to track individuals and so to estimate rates of completion of these “additional” registrants with other registrants. However, the changes to LLTTF registration procedures and the difficulties of trying to match IPs from one website to another using time of day made this impractical. LLTTF currently ask participants for their full postcode which probably deters some from giving any information and may encourage falsification or error. We have recommended changes to data collection methods in LLTTF, asking for postcode area from a drop down list. Analytics location information would be more useful if the area associated with a town name was transparent to users, and if it could be aligned with population figures. We have tried to suggest to Google that their geography should be changed. In the meantime, the geography used by Google for London and immediate surroundings suggest that, to have more accurate location data in a study, London should be excluded.

#### Finding a Period of Website Stability

Studies that aim to change Internet use will be limited by frequent changes to websites and other technical advances. In our case, even though CW was the author of LLTTF, changes underway to the LLTTF website could not be postponed for this study. Our sample was selected based on data extracted from LLTTF between the years 2008 and 2009 when most users of the site registered. In January 2011, after our study had been designed and ethical approval had been sought, LLTTF was reconfigured such that registration was optional and could be done at a later time. As a result, the number of people that registered greatly reduced. This meant that registration figures were no longer directly comparable with those collected earlier. Although we were able to compare the relative differences between regions in 2009 with 2011, by creating an index of use based on the lowest use region, we restricted direct comparison (ie, the “before” period) to January- April 2011.

#### Demographics

Graham [18] found that online advertising could be an effective and cost-efficient strategy to reach and engage Spanish-speaking Latino smokers in an evidence-based Internet cessation program. She concluded that cultural targeting and smoking-relevant images might be important factors for banner advertisement design. In this pilot we did not collect demographic details. These were collected by the target LLTTF website as part of registration and we hope to achieve better record linkage in the future.

#### Sample Selection

In this pilot study we selected our sample trying to match for the populations of postcode areas and the baseline registration rates on LLTTF. Although it would be important not to have a grossly imbalanced sample, the need for a greater number of postcode areas (as described above) while ensuring geographical dispersion to avoid contamination would seem to be more important in the design.

### Comparison Against Other Methods

Although various online methods exist to raise awareness, the problem of being able to select sample areas allowing for the competing demands of contamination and confounding factors suggest that having a factorial design with more than one recruitment intervention would be difficult. On the other hand, for practitioners seeking to increase recruitment using a mix of all possible methods, more than one recruitment intervention would seem to be sensible.

### Conclusions for Practitioners and Policy Makers

Our pilot study confirms other research that many people search online for help with mental health issues [2] such as depression; our advert was displayed 673,074 times in just over 7 months for a total targeted population of 3.5 million. Anecdotally, in discussing this research, many practitioners responded by saying “I never click on adverts” but our pilot has shown that nearly 8000 people clicked on our short advert for NHS recommended sites and many of these were depressed, according to their answers on a self-completed questionnaire.

In discussing this research with NHS policy makers, the response to advertising, perhaps in the light of previous criticisms of their expenditure, was that NHS websites have been optimized and therefore appear high in search results, so there is no need for online advertising. This may be true but our exploration of this [34] suggested that even though online CBT sites can eventually be found via NHS and Royal College of Psychiatrists websites that tended to appear high in search results, the probability was low, and was significantly increased for a naïve user by the addition of an advert. That evidence however was theoretical and the only way of knowing for sure about the cost-effectiveness of online advertising was to study it in a location targeted RCT.

Although the results of the weblinks in this pilot study was not as expected and will be excluded from this research design (unless a better method of placing local weblinks can be found), it would be wrong to conclude that weblinks are not relevant for practitioners and policy makers. The problem may be that the type of site containing relevant links tends to be either national (inappropriate for a location targeted clusters RCT) or very local (researchers likely do not have prior knowledge about existence of these sites). Practitioners and policy makers may therefore have to rely on weaker evidence from before/after studies of cost and impact to decide on how much effort they put into using weblinks.

Part of the reason why LLTTF had greater use in Scotland compared to other parts of Britain is that professionals in those areas were more aware of the existence of these sites and recommended use of these sites to patients. The role of professionals in recommending online CBT may also have explained the confounding results seen in one area in the control group. So it may be that continuing professional development and raising awareness among professionals about resources for depression may be as, or more effective, than direct-to-patient online interventions. However only one third of patients were in contact with health services and other studies have shown that trying to reach patients to tell them about online resources may be labour intensive, time consuming, and very expensive [24]. Further research is needed to compare the cost-effectiveness of improving access via professionals versus direct to patient methods, but in the meantime, practitioners and policy makers should keep online advertising as an option.

### Conclusions for Researchers

#### Overall Conclusions

A cluster geo-located RCT to test the cost-effectiveness of online advertising seems feasible in Britain. Geolocated adverts are offered by Google in other countries based on a radius around a point, so the general design of a cluster RCT to test the cost effectiveness of online advertising in raising awareness of an online therapy, as piloted in this study, would seem to hold true for other countries and for other online therapies or websites. However, this pilot study has demonstrated 4 general messages concerning contamination, confounding, dose of advertising, and length of study that will be useful for other researchers.

#### Contamination

Provided it is possible to have a sufficiently large buffer zone between intervention and control regions, it should be possible to deal with potential contamination. The definition of sufficiently large is vague and needs to be piloted in each country, but in Britain, we think a 2-arm trial with 16 postcode areas in each should be possible without too much contamination. The problems of including London in Britain were described in more detail elsewhere [10]. It seems likely that similar studies in other countries may need to exclude the capital or major centres for Internet providers. We have also previously described other design issues with using AdWords including the need for separate campaigns for each postcode area [10].

#### Confounding Factors

Ideally, such a trial would have a large number of clusters in each arm such that any one region is kept to approximately 5% of the total population in the arm. That would mean that any confounding activity such as local media campaigns would not influence the arm greatly. However, dealing with confounding factors conflicts, to some degree, with the relatively small and densely populated country like Britain. In our proposed best design for Britain, trying to keep contamination to a minimum at the postcode area level, our design of 2-arms of 16 postcode areas each, still included a city representing 17% of that arm. This was slightly risky for introducing confounding factors but was the best we could do. In a bigger country such as the United States, it should be possible to have a stronger design. In a smaller, densely populated country such as the Netherlands, this study design may be impossible.

#### Dose of Advertising

We recommend that in such a design, adverts are linked to a special “landing page” within the target site. This allows those following the link to track their use of the target site. We had used an intermediate project website in this pilot because we were offering 2 online CBT sites and 2 other sites. This gave us considerable problems in trying to track participants from the advert to the project website to the target website, and in trying to match up the different data sets available from AdWords, Analytics, and website logs. Having a landing page within the target website should remove most problems but it would still help in the design if the dose of advertising, and so the expected “footfall” from advertising was substantial compared to the number arriving from other sources. In this pilot, with a limited advertising budget, although we had significant numbers arriving at our project website, the problem of leakage meant that the number of people finding the target website compared to the numbers finding it from a normal search, national weblinks, or direct entry were small. Any study with this type of design should pay greater attention to the total footfall on the target website and try to calculate the dose of advertising needed to have a demonstrable impact.

#### Length of Study

The cost of advertising in such a trial will be partly determined by how long the campaign is run. We ran our pilot for 7.5 months. Although it did take several weeks for our campaign to stabilize and for AdWords to get the best return and cost per click, a campaign of 4-6 months should be sufficient for this type of study where the total number clicking is in the range of 300-500 people. The best design (as described above) is to have a larger dose of advertising over a shorter period rather than a small dose over a longer period.

### Limitations

We cannot be sure how the findings of this study would translate to other countries and there is no guarantee that location targeting of online advertising will continue to be available in this form. Increasing use of mobile phones may change the way location targeted adverts work. AdWords is of course not the only way of local advertising online and a definitive trial might consider use of advertising solutions from Microsoft, Facebook, LinkedIn, and others. Facebook, for example, offers location targeting and would also be worth exploring in this way.

Our study was limited by the difficulties of trying to match different data sources. Different sources from Google (AdWords and Analytics) do not exactly match due to different ways of collecting the data. Other issues, because of the anonymity of the data, include whether visitors are unique individuals. For example, although Analytics may claim to report unique visitors, we cannot verify that claim and it would be impossible for Analytics to differentiate between two individuals using the same computer (IP address) and one person with two emails using the same computer. LLTTF only collects emails of those who register. It is likely, therefore, that all sources overestimate the number of unique individuals. However, although numbers from different sources do not match exactly, the overall picture seems consistent and reasonably robust.

### Overall Conclusion

This pilot study has shown that a definitive cluster trial of AdWords is worthwhile, and that this type of design could be used to assess other online recruitment interventions.

## Multimedia Appendix 1

Screenshots from Online Help For Depression.

## Multimedia Appendix 2

Two arms for definitive cluster randomized trial, showing the 16 postcode areas in each arm, cluster populations, and total population for each arm.

## Abbreviations

CBT: cognitive behavioral therapy
GP: general practitioner
IAPT: Improved Access to Psychological Therapies
IP: Internet protocol
KT: Kingston
LLTTF: Living Life To The Full
NHS: National Health Service
NICE: National Institute for Health and Clinical Excellence
OHFD: Online Help For Depression
PHQ9: Patient Health Questionnaire
RCT: randomized controlled trial
