# Supplementary figures and images for: Recruitment to Online Therapies for Depression: Pilot Cluster Randomized Controlled Trial
Source: J Med Internet Res. 2013 Mar 5;15(3):e45. doi: 10.2196/jmir.2367 (PMC3636297; doi:10.2196/jmir.2367)

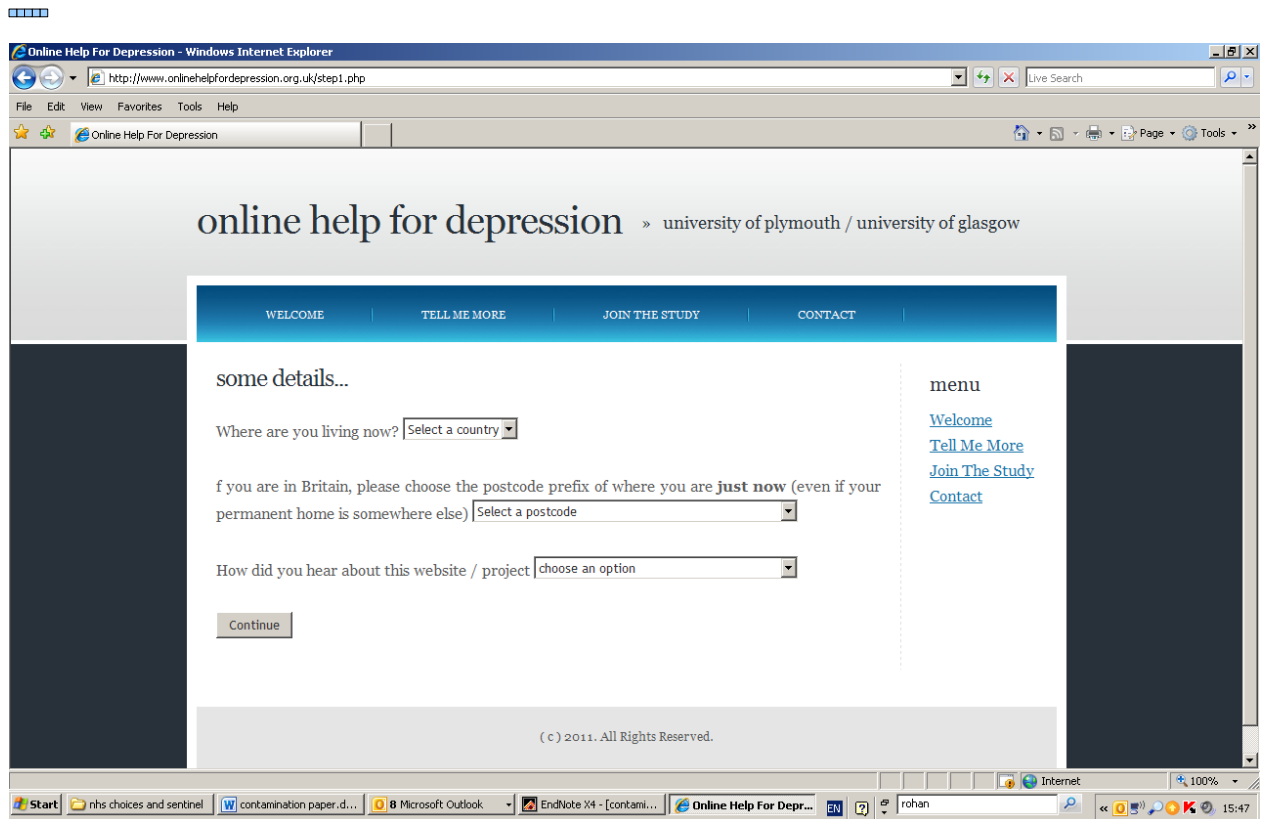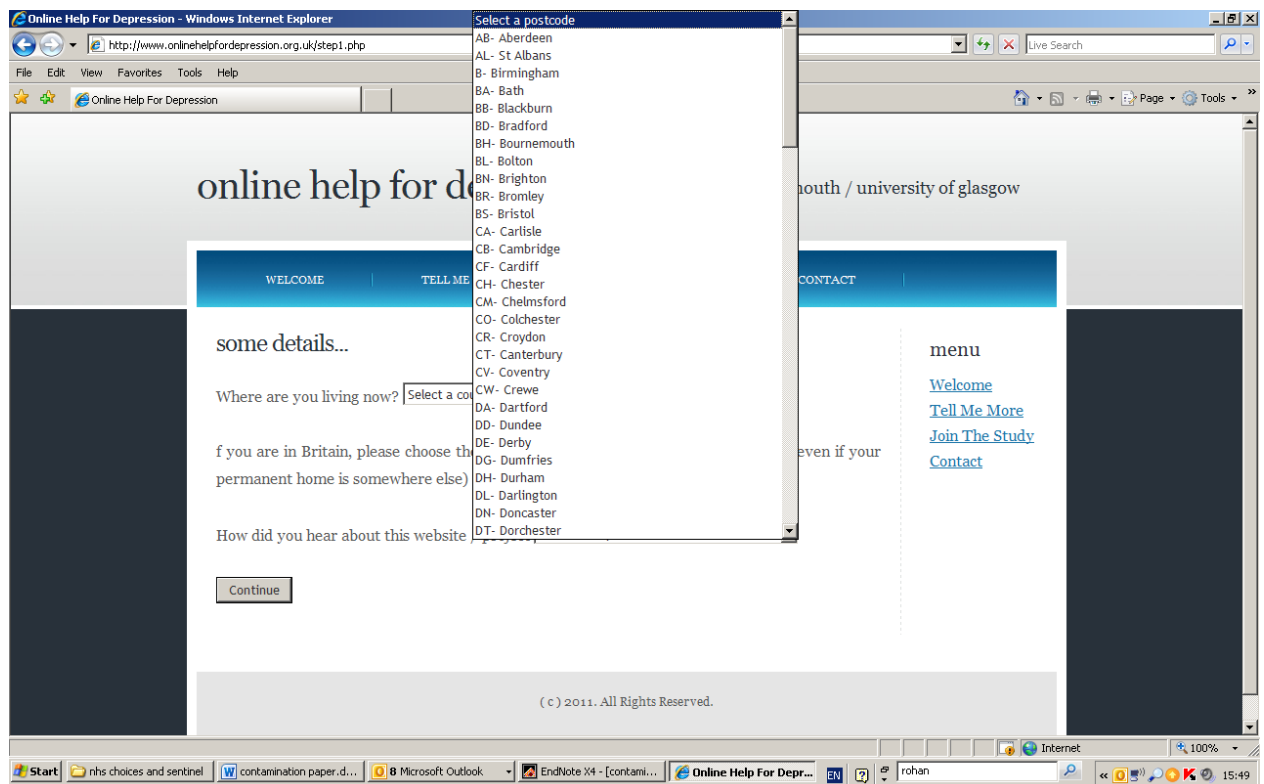

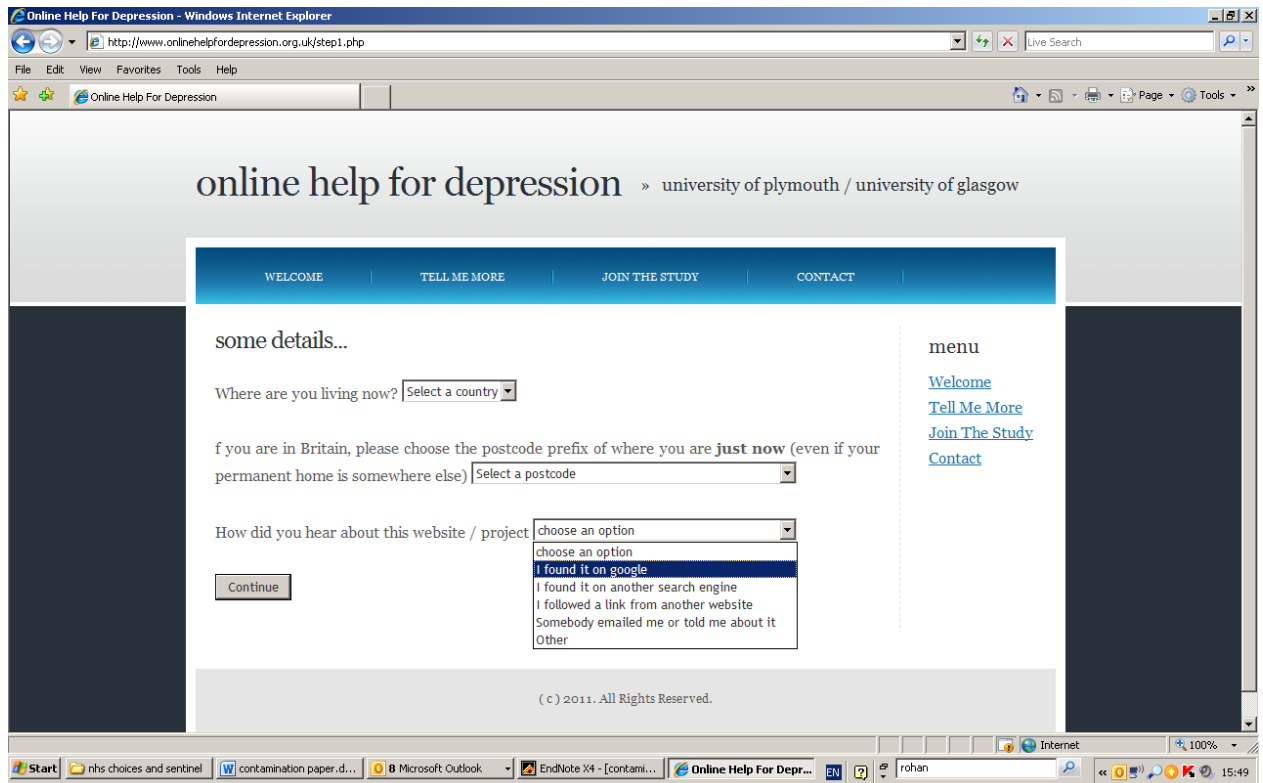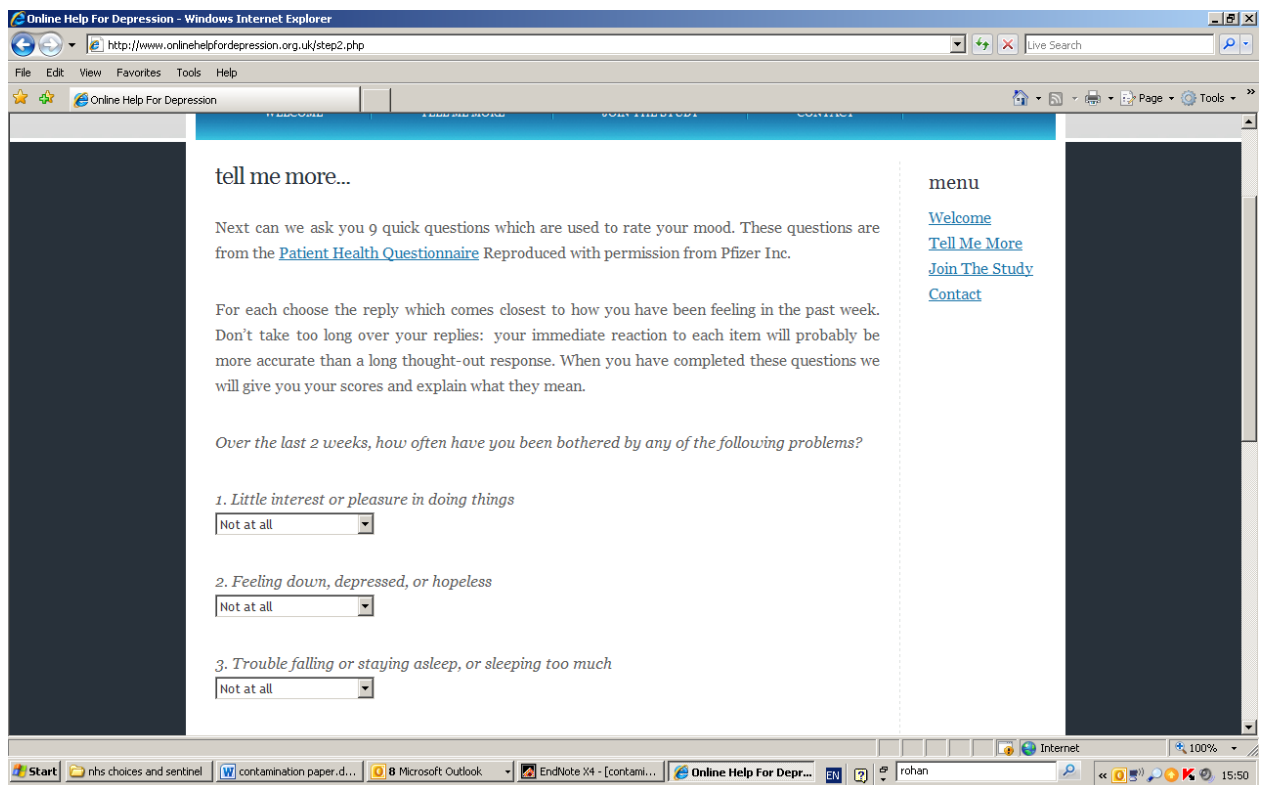

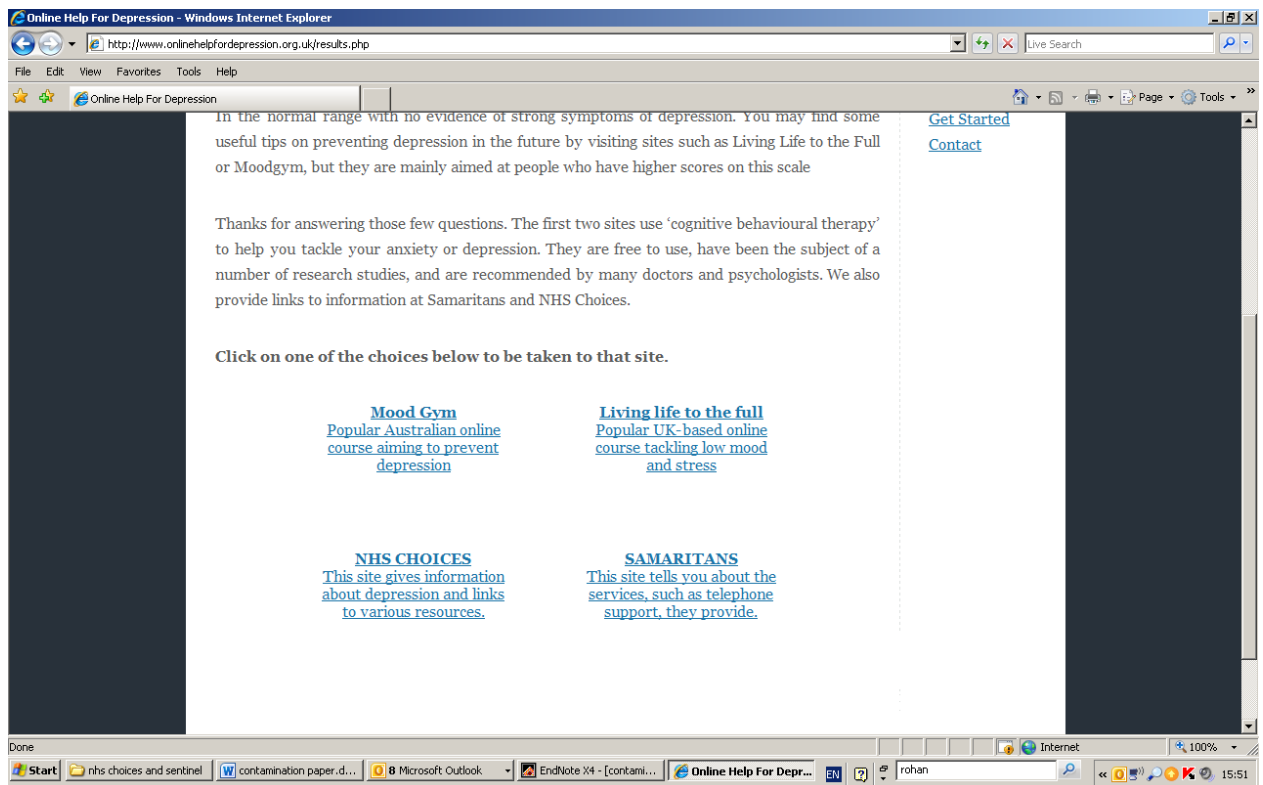

Supplement: Supplementary file 1 [file jmir_v15i3e45_app1.pdf]
