# Supplementary material for: Recruitment to Online Therapies for Depression: Pilot Cluster Randomized Controlled Trial
Source: J Med Internet Res. 2013 Mar 5;15(3):e45. doi: 10.2196/jmir.2367 (PMC3636297; doi:10.2196/jmir.2367)
Supplement: Supplementary file 2 [file jmir_v15i3e45_app2.pdf]

Appendix 1

Arm 1			Arm 2		
1,766,297	Birmingham	B	1,284,043	Sheffield	S
1,121,690	Newcastle upon Tyne	NE	1,164,515	Glasgow	G
1,026,115	Manchester	M	936,498	Cardiff	CF
859,835	Bristol	BS	893,369	Leicester	LE
843,450	Liverpool	L	810,212	Edinburgh	EH
737,343	Leeds	LS	774,149	Portsmouth	PO
729,642	Reading	RG	629,029	Tonbridge	TN
605,362	Chelmsford	CM	619,948	Stoke-on-Trent	ST
584,976	Cleveland	TS	517,016	Llandudno	LL
490,104	Kingston upon Thames	KT	510,223	Plymouth	PL
477,813	Blackburn	BB	461,830	Aberdeen	AB
420,913	Harrow	HA	422,339	Hull	HU
309,400	Luton	LU	273,437	Blackpool	FY
200,471	Dorchester	DT	265,416	Lincoln	LN
167,155	Hereford	HR	147,625	Dumfries	DG
21,988	Shetland	ZE	26,502	Hebrides	HS
Total population 10,362,554<br>Assume 5% depression 518,128			Total population 9,731,637<br>Assume 5% depression 486,582		
